# Protein breakdown in muscle wasting: Role of autophagy-lysosome and ubiquitin-proteasome^☆^^☆☆^

**DOI:** 10.1016/j.biocel.2013.04.023

**Published:** 2013-10

**Authors:** Marco Sandri

**Affiliations:** a Venetian Institute of Molecular Medicine (VIMM), Padova, Italy; b Consiglio Nazionale delle Ricerche (CNR) Institute of Neuroscience, Padova, Italy; c Department of Biomedical Sciences, University of Padova, Padova, Italy

**Keywords:** Skeletal muscle, Atrophy, Autophagy, Ubiquitin protesaome, FoxO, Muscle wasting

## Abstract

Skeletal muscle adapts its mass as consequence of physical activity, metabolism and hormones. Catabolic conditions or inactivity induce signaling pathways that regulate the process of muscle loss. Muscle atrophy in adult tissue occurs when protein degradation rates exceed protein synthesis. Two major protein degradation pathways, the ubiquitin-proteasome and the autophagy-lysosome systems, are activated during muscle atrophy and variably contribute to the loss of muscle mass. These degradation systems are controlled by a transcription dependent program that modulates the expression of rate-limiting enzymes of these proteolytic systems. The transcription factors FoxO, which are negatively regulated by Insulin-Akt pathway, and NF-κB, which is activated by inflammatory cytokines, were the first to be identified as critical for the atrophy process. In the last years a variety of pathways and transcription factors have been found to be involved in regulation of atrophy. This review will focus on the last progress in ubiquitin-proteasome and autophagy-lysosome systems and their involvement in muscle atrophy. This article is part of a Directed Issue entitled: Molecular basis of muscle wasting.

## 1 Introduction

Skeletal muscle mass and muscle fiber size may vary according to physiological and pathological conditions. An increase in muscle mass and fiber size, i.e. muscle growth or hypertrophy, occurs during development, in response to mechanical overload and/or anabolic hormonal stimulation. During development, the growth of skeletal muscle, like the mass of any other tissue, depends on protein and cellular turnover. In adulthood, the regulation of muscle mass and fiber size essentially reflects *protein turnover*, namely the balance between protein synthesis and degradation within the muscle fibers. Muscle atrophy involves the shrinkage of myofibers due to a net loss of proteins, organelles and cytoplasm. Acute muscle atrophy, as occurs in many pathological conditions, is due to hyperactivation of the cellular main degradation pathways, including the ubiquitin-proteasome system and the autophagy-lysosome pathways. Recent studies have highlighted a complex scenario whereby these catabolic signaling modulate one another at different levels, and are also connected at various levels to biosynthetic pathways (Bonaldo and Sandri, 2013). The result is a coordinated balance between protein degradation and synthesis that reflects the physiological state of the muscle fiber. Before considering mechanisms of muscle atrophy, it is useful to make some general considerations:

(1) A transcriptional dependent upregulation of the atrophy-related gene at mRNA level does not always match with an increase of protein level. The field of muscle wasting should consider the fact that transcriptional-dependent induction of several atrophy related genes is required to replenish the enzymes/proteins that are lost during the enhanced protein breakdown. Importantly, the muscle-specific atrophy-related ubiquitin ligases atrogin1/MAFbx and MuRF1 similarly to other E3s, undergo auto-ubiquitination (Bodine et al., 2001). It is reasonable that an increased ligase activity of these E3s during inactivity or catabolic conditions would inevitably amplify their auto ubiquitination action, thus resulting in increased proteasomal-dependent degradation. Therefore, the transcriptional upregulation is particularly important mostly to replenish the loss of the ubiquitin ligase protein that occurs as a consequence of the increased activity. This concept should be considered when protein expression does not match with transcript induction. In autophagy-lysosome system, the proteins LC3, p62 and BNIP3 are critical for membrane commitment, cargo delivery and selective removal of damaged mitochondria, respectively. To elicit their functions these proteins are entrapped into the autophagosome when the vesicle is formed and therefore, are destroyed upon fusion of autophagosome with lysosome. The transcriptional dependent upregulation of these genes is important to maintain their level during an enhancement of autophagy flux.
(2) A decrease of protein synthesis can not be considered alone as the mechanism of atrophy. The size of a postmitotic cell results from the balance between protein synthesis and degradation. Indeed, in condition of protein synthesis inhibition the total protein content of the cell is affected by protein’ half-life that depends on basal protein breakdown. Therefore, paradoxically in situations of decreased protein synthesis the cell size ultimately depends more on protein breakdown than in circumstances of normal protein synthesis.
(3) A decrease of nuclear turnover or of the satellite cell pool can not explain muscle atrophy in adulthood.The myonuclear domain is not constant in various conditions including growth and atrophy. Recent evidences underlie that myonuclear turnover is negligible in adulthood after postnatal growth (White et al., 2010). Moreover, no myonuclear addition has been shown in different models of muscle hypertrophy and depletion of satellite cells does not prevent muscle hypertrophy (Amthor et al., 2009; Blaauw et al., 2009; Jackson et al., 2012; Lee et al., 2012; Raffaello et al., 2010). In addition, reduction of Pax7 positive cells in adult muscles is not sufficient to trigger muscle wasting in normal condition (McCarthy et al., 2011). These recent findings together with the concept that during muscle atrophy the myonuclear domain decreases suggest that lack of satellite cell proliferation and fusion is not the major contributor of the atrophy process.

## 2 Muscle atrophy

Muscle atrophy involves the shrinkage of myofibers due to a net loss of proteins, organelles and cytoplasm. The ubiquitin-proteasome system and the autophagy-lysosome pathway are the degradation systems involved in this process.

### 2.1 The autophagy-lysosome system

From the discovery that FoxO transcription factors coordinate the activation of the ubiquitin proteasome and of autophagy-lysosome pathways in atrophying muscles (Sandri, 2010), a great interest emerged for autophagy and many studies have underlined the induction of this system in different pathological conditions of muscle wasting. A detailed description of the different types of autophagy, including their regulation and involvement in muscle homeostasis has been recently reviewed (Bonaldo and Sandri, 2013). Briefly, autophagy is a highly conserved homeostatic mechanism used for the degradation and recycling, through the lysosomal machinery, of bulk cytoplasm, long-lived proteins and organelles (Mizushima and Komatsu, 2011). Although autophagy was initially considered a non-selective degradation pathway, the presence of more selective forms of autophagy is becoming increasingly evident. Indeed, autophagy can trigger the selective removal of specific organelles, such as mitochondria via mitophagy or protein aggregates.

Several years ago autophagy-lysosome system was described to be activated in muscle cells during catabolic conditions (Bechet et al., 2005; Deval et al., 2001; Tassa et al., 2003). However, the interest on this pathway emerged recently in the muscle community. During the last few years autophagy has been found to be modulated in muscle by a plethora of situations including cancer (Penna et al., 2013), ageing (Penna et al., 2013; Wenz et al., 2009; Wohlgemuth et al., 2010), fasting (Mammucari et al., 2007; Mizushima et al., 2004), caloric restriction (Grumati et al., 2010; Wohlgemuth et al., 2010), sepsis (Mofarrahi et al., 2012), critically illness (Derde et al., 2012), cirrhosis (Qiu et al., 2012), chemiotherapy (Smuder et al., 2011), disuse (Brocca et al., 2012) and denervation (O’Leary et al., 2012a; Zhao et al., 2007). Importantly, autophagy is physiologically induced by exercise including both endurance and resistance exercise and mediates the metabolic beneficial effects of physical activity on glucose homeostasis (Grumati et al., 2011; He et al., 2012; Jamart et al., 2012a,b; Luo et al., 2013). We were the first to discover that autophagy is activated by endurance exercise (Grumati et al., 2011) and this effect have been recently confirmed on humans (Jamart et al., 2012a,b). The rationale of exercise-dependent autophagy activation is still unclear but evidences suggest that autophagy is important for removal of proteins/organelles that are damaged by exercise itself or is a mechanism to provide energy for sustained contraction. These two hypotheses need to be better defined soon. For instance, the initial observation that autophagy is critical for glucose homeostasis has been recently challenged. In fact, opposite results have been recently published related to the role of autophagy on insulin sensitivity in skeletal muscle. Indeed both activation or inhibition of autophagy have been reported to ameliorate glucose uptake and lipids metabolism in diet-induced obesity (He et al., 2012; Kim et al., 2013). The two studies have used different transgenic mice that may account for the different results. The initial study had used transgenic mice that contain knock-in mutations in BCL2 phosphorylation sites that prevent stimulus-induced disruption of the BCL2-beclin-1 complex and autophagy activation (He et al., 2012). These mice are characterized by a normal autophagy flux that cannot be increased by fasting or exercise. BCL2 mutant mice, which express this mutant protein in every tissue, show decreased endurance and altered glucose metabolism during acute exercise, as well as impaired chronic exercise-mediated protection against high-fat-diet-induced glucose intolerance. In this study the cell autonomous issue was never addressed therefore, we do not have any idea how and which tissue contributes to the beneficial effect on sugar homeostasis. The second study used a muscle-specific Atg7 knockout mice in which autophagy is selectively blocked in skeletal muscle. These animals showed decreased fat mass and were protected from high fed diet (HFD) induced obesity and insulin resistance (Kim et al., 2013). The amelioration of insulin sensitivity is due to a mitochondrial-dependent signal. In fact mitochondrial dysfunction induced by autophagy deficiency stimulated Fgf21 expression through activation of Atf4, a transcription factor activated by the Unfolded Protein Response pathway. Fgf21 is released from muscle and its inhibition attenuates the beneficial effects on glucose tolerance and insulin sensitivity of muscle-specific autophagy knockout during diet-induced obesity.

**Fig. 1:**
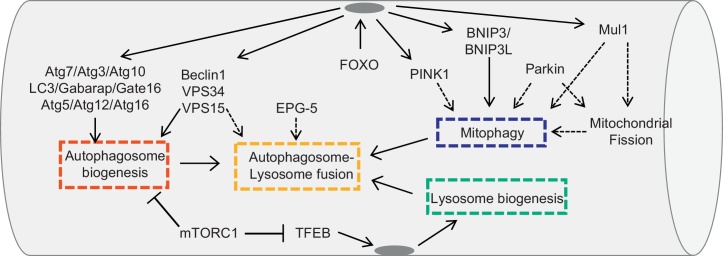
Signaling modules responsible for autophagy-lysosome pathway in the adult. The different modules are: autophagosome formation, selective autophagic removal of mitochondria, autophagosome docking and fusion with lysosome and new lysosome formation. Each module is controlled by different pathways and factors. The dotted lines point to mechanisms that are not yet known.

Mitochondria regulation is important to preserve muscle function and for the regulation of general metabolism and indeed, this organelle changes morphology and number as consequence of muscle activity. The mitochondrial network can be remodeled by the fusion/fission proteins, the mitochondrial shaping machinery, and by the selective removal of small mitochondria via autophagy, a process named mitophagy (Romanello and Sandri, 2012) (Fig. 1). In mammals, parkin, PINK1, Bnip3 and Bnip3L have been shown to regulate mitophagy, and inactivation of the genes coding for these proteins leads to mitochondrial abnormalities (Bothe et al., 2000; Hara et al., 2006). PINK1 is normally absent in healthy mitochondria because it is constitutively degraded by mitochondrial proteases. However, once mitochondria are damaged, PINK1 is no longer degraded and accumulates. Moreover, PINK1 is also under transcriptional control of FoxO family (Fig. 1) PINK1 induces parkin recruitment to mitochondria, promoting mitophagy through ubiquitination of outer mitochondrial membrane proteins that are recognized by p62, which then brings autophagic vesicles to ubiquitinated mitochondrial proteins (Narendra and Youle, 2011; Youle and Narendra, 2011). Parkin also ubiquitinates mitofusins promoting their degradation via proteasome (Sarraf et al., 2013; Ziviani et al., 2010). This action is important to prevent mitochondrial fusion and to facilitate mitochondrial fragmentation and removal via mitophagy. Bnip3 and Bnip3L are BH3-only proteins localized at the outer membrane of the mitochondria after cellular stress, and bind directly to LC3, thereby recruiting the autophagosome to damaged mitochondria (Hanna et al., 2012; Novak et al., 2010). In atrophying muscle, the mitochondrial network is dramatically remodeled following fasting or denervation, and autophagy via Bnip3 contributes to mitochondrial changes (Mofarrahi et al., 2012; O’Leary et al., 2012b; Romanello et al., 2010; Romanello and Sandri, 2010). Expression of the fission machinery is sufficient to cause muscle wasting in mice, whereas inhibition of mitochondrial fission prevents muscle loss during denervation, indicating that disruption of the mitochondrial network is a crucial amplificatory loop of the muscle atrophy program (Romanello et al., 2010; Romanello and Sandri, 2010). Conversely, impairment of basal mitophagy leads to the accumulation of damaged and dysfunctional mitochondria that contribute to myofiber degeneration (Grumati et al., 2010). Therefore autophagy is critical to maintain myofiber function by clearing abnormal organelles. Accordingly, the phenotype of mice with muscle-specific inactivation of various genes coding for autophagy-related proteins, such as Atg7, Atg5 or nutrient-deprivation autophagy factor-1 (NAF-1), a Bcl-2 associated autophagy regulator, results in atrophy, weakness and different myopathic features (Chang et al., 2012; Masiero et al., 2009; Raben et al., 2008). In addition, altered regulation of autophagy-related genes leads to muscle dysfunction. Histone deacetylases 1 and 2 (HDACs) were found to regulate muscle autophagy by controlling the expression of autophagy genes. Muscle-specific ablation of both HDAC1 and HDAC2 results in partial perinatal lethality, while those HDAC1/2 knockout mice that survive develop a progressive myopathy characterized by impaired autophagy (Moresi et al., 2010, 2012).

Despite the important advancement in signaling pathways that control the autophagy machinery, the precise functions, in vivo, of the core autophagy components in membrane commitment, growth and fusion to form an autophagosome as well as in the docking of autophagosome to lysosome and in the fusion into an autophagolysosome are largely unknown. One of the most important regulatory element is the beclin1/Vps34/Vps15 complex. Vps34 is a Class III phosphatidylinositol 3-kinase (PI3K) enzyme that generates PI3-phosphate from PI, while Vps15 is a regulatory subunit that is required for Vps34 activity. Therefore, Vps34 and Vps15 are obligate partners that form the core for the association of different proteins to generate distinct complexes that regulate autophagosome biogenesis and endosome trafficking. The beclin1/Vps34/Vps15/Atg14L complex is required for autophagosome biogenesis and indeed drugs that block Vps34 activity prevents autophagosome formation in cell culture. The role of Vps15 in adult muscle has been recently investigated by a loss of function approach. Surprisingly, muscle-specific Vps15 knockout mice showed normal LC3 lipidation and autophagosome formation but impaired autophagosome lysosome fusion (Nemazanyy et al., 2013). Importantly, deletion of Vps15 results in almost disappearance of Vps34 and beclin1 proteins suggesting that these proteins are unstable when they do not interact to form a complex. Therefore, the first important finding is that in adult tissue the PI3K complex is not required for the correct function of the autophagy conjugation system and for autophagosome biogenesis but its action is more relevant in autophagosome docking and fusion with lysosomes (Fig. 1). The second important finding is related to muscle phenotype. In fact Vps15 knockout mice showed myopathic features including buildup of autophagosomes, abnormal glycogen accumulation, anomalous lysosomes, necrotic cell death, regenerating myofiber and elevated creatine kinase plasma levels. These characteristics are reminiscent of Autophagic Vacuolar Myopathy. Importantly, overexpression of Vps15 and Vps34 reactivates autophagy and cleared glycogen accumulation in myotubes obtained from Danon patient, a lysosomal storage disease characterized by glycogen buildup. These findings, accordingly to recent data on Pompe patients (Nascimbeni et al., 2012a,b), suggest that autophagy impairment is the major pathogenetic mechanism that contributes to muscle wasting in lysosome storage disorders and that autophagy reactivation is beneficial and not detrimental for myofiber function. This concept is further supported by the first genetic disease casued by a mutation in an autophagy-related gene, the Vici syndrome. Vici syndrome is a recessive inherited multisystem disorder characterised by cardiomyopathy, callosa agenesis, cataracts, and combined immunodeficiency. Interestingly, skeletal muscle of Vici patients display consistent myopathic features, including atrophy of type 1 fibers, centrally nucleated fibers and abnormal glycogen accumulation (Cullup et al., 2013). Electron microscopy analyses of myofibers revealed the presence of exocytic vacuoles, numerous vacuole-like area and dense bodies that resemble lysosomes and abnormal mitochondria. The genetic defect has been identified in mutations of EPG5 gene, which encodes for a protein predominantly expressed in cardiac and skeletal muscle, central nervous system, thymus, immune cells, lung and kidney. Importantly, the *C. elegans* homolog of EPG-5 plays a critical role in the autophagy process (Tian et al., 2010) (Fig. 1). In agreement with worms, myofibers and fibroblast of Vici patients display accumulation of p62, Nbr1 and lipidated LC3, thus confirming that the autophagy system is blocked (Cullup et al., 2013). The presence of LC3/p62 and Nbr1/p62 double positive puncta, as well as the reduction of colocalization between LC3 and LAMP-1, suggest that the fusion of autophagosome with lysosome is blocked in these patients (Cullup et al., 2013). These data have been confirmed by the phenotype of EPG-5 knockout mice. Deletion of EPG-5 leads to selective damage of cortical layer 5 pyramidal neurons and spinal cord motor neurons resulting in muscle degeneration, myofiber atrophy, and reduced survival (Zhao et al., 2013). Interestingly, morphological studies of gastrocnemius muscle showed centrally nucleated and vacuolated fibers, while ultrastructural analyses revealed misalignment of Z-lines and accumulation of abnormal enlarged mitochonria resembling the features of Atg7 deficient muscle. Consistently with Vici patients, autophagy flux is blocked in gastrocnemius muscle of EPG-5 knockout mice. An interesting observation is that deletion of EPG-5 leads to abnormalities in endosome trafficking and receptor recycling. Therefore, Vici syndrome is the first multisystemic disorder associated with defective autophagy. The role of EPG5 in the autophagy pathway and the crass talk between autophagy and endocityc system are two important points that need to be explored in the future.

### 2.2 The ubiquitin-proteasome

In the ubiquitin-proteasome system, proteins are targeted for degradation by the 26S proteasome through covalent attachment of a chain of ubiquitin molecules. The ubiquitin ligase enzyme, or E3, binds the protein substrate and catalyzes the movement of the ubiquitin from the E2 enzyme to the substrate. This is the rate-limiting step of the ubiquitination process, which affects the subsequent proteasome-dependent degradation. Once the protein is ubiquitinated it is docked to the proteasome for degradation, unless the polyubiquitin chain is removed by the de-ubiquitinating enzymes. Among the different E3s, only a few have been found to regulate atrophy process and to be transcriptionally induced in atrophying muscle.

The first to be identified were Atrogin-1/MAFbx and MuRF1. These two E3s are specifically expressed in striated and smooth muscles (Bdolah et al., 2007; Bodine et al., 2001; Gomes et al., 2001). Atrogin-1/MAFbx and MuRF1 knockout mice are resistant to muscle atrophy induced by denervation (Bodine et al., 2001). MuRF1 knockout mice are also resistant to dexamethasone-induced muscle atrophy (Baehr et al., 2011) while, knockdown of atrogin-1 spare muscle mass in fasted animals (Cong et al., 2011). Thus far, MuRF1 ubiquitinates several muscle structural proteins, including troponin I (Kedar et al., 2004), myosin heavy chains (Clarke et al., 2007; Fielitz et al., 2007), actin (Polge et al., 2011), myosin binding protein C and myosin light chains 1 and 2 (Cohen et al., 2009). Whereas the substrates of atrogin-1 that have been identified seem to be involved in growth-related processes or survival pathways. Atrogin-1 promotes degradation of MyoD, a key muscle transcription factor, and of eIF3-f, an important activator of protein synthesis (Csibi et al., 2010; Tintignac et al., 2005). In the heart, atrogin-1 ubiquitinates and reduces the levels of calcineurin A, an important factor triggering cardiac hypertrophy in response to pressure overload (Li et al., 2004). However a recent work, have found that atrogin-1 interacts with sarcomeric proteins, including myosins, desmin, and vimentin, as well as transcription factors, components of the translational machinery, enzymes involved in glycolysis and gluconeogenesis and mitochondrial proteins (Lokireddy et al., 2012a). Whether this interaction results in ubiquitination of these proteins has yet to be proven.

Since two ubiqutin ligases cannot effort for the degradation of all the sarcomeric and soluble proteins, additional E3s are involved in muscle loss. Specific ubiquitin-ligases may be involved in different models of muscle wasting and at different stages of the atrophy process. For instance, the HECT domain ubiquitin ligase Nedd4-1 has been reported to be upregulated mainly during disuse. Indeed deletion of the Nedd4-1 gene specifically in skeletal muscle results in partial protection from muscle atrophy in denervated type II fibers. However, Nedd4-1 knockout mice have smaller muscles, suggesting that this E3 may play additional roles during myogenesis or in the control of protein synthesis (Nagpal et al., 2012). Recently, Trim32 has been reported to degrade thin filaments (actin, tropomyosin and troponins), α-actinin and desmin (Cohen et al., 2012). However, Trim32 knockout mice are not protected from atrophy, but instead show an impaired recovery of muscle mass after atrophy (Kudryashova et al., 2012).

Another E3 ubiquitin ligase found to play a critical role in atrophy is TRAF6 (Paul et al., 2010), which mediates the conjugation of Lys63-linked polyubiquitin chains to target proteins. Lys48-linked polyubiquitin chains are a signal for proteasome-dependent degradation, but Lys63-linked polyubiquitin chains play other roles, such as regulating autophagy-dependent cargo recognition by interacting with the scaffold protein p62 (also known as SQSTM1) (Kirkin et al., 2009; Komatsu et al., 2007; Pankiv et al., 2007). Notably, muscle-specific TRAF6 knockout mice have a decreased amount of polyubiquitinated proteins, almost no Lys63-polyubiquitinated proteins in starved muscles (Paul et al., 2012) and are resistant to muscle loss induced by denervation, cancer or starvation (Kumar et al., 2012; Paul et al., 2010, 2012). The mechanism of this protection involves both direct and indirect effects of TRAF6 on protein breakdown. Inhibition of TRAF6 reduces the induction of atrogin-1 and MuRF1, thereby preserving muscle mass under catabolic conditions. Moreover, TRAF6-mediated ubiquitination may have additional function on modulating intracellular signaling. In fact TRAF6 is required for the optimal activation of JNK, AMPK, FoxO3 and NF-κB pathways (Paul et al., 2012). The effects on FoxO3 and NF-κB may explain why atrogin-1 and MuRF1 are less induced in TRFAF 6 knockout mice.

Mul1 is a mitochondrial ubiquitin ligase that plays an important role in mitochondrial network remodeling. Mul1 is upregulated in catabolic conditions, such as fasting or denervation, by the FoxO family of transcription factors and causes mitochondrial fragmentation and removal via autophagy (Lokireddy et al., 2012b). Importantly, knocking down Mul1 spares muscle mass during fasting. It is unclear whether such protection occurs in other models of muscle wasting such as denervation. Mul1 ubiquitinates the mitochondrial pro-fusion protein mitofusin 2, causing its degradation via the proteasome system. The exact mechanism that triggers Mul1-dependent mitochondrial dysfunction and mitophagy is unclear (Fig. 1), but it has been reported that mitofusin degradation is permissive for mitochondrial fission and mitophagy (Romanello et al., 2010).

Another ligase is CHIP, which regulates ubiquitination and lysosomal-dependent degradation of filamin C, a muscle protein found in the Z-line (Arndt et al., 2010). Filamins undergo unfolding and refolding cycles during muscle contraction and are therefore prone to irreversible damage (Arndt et al., 2010). Alterations to filamin structure triggers the binding of the co-chaperone BAG3, which carries a complex made up of the chaperones Hsc70 and HspB8, as well as the ubiquitin ligase CHIP. CHIP ubiquitinates BAG3 and filamin, which are recognized and delivered to the autophagy system by p62 (Arndt et al., 2010). Interestingly, filamin B is controlled, at least during myogenesis, by another ubiquitin ligase, ASB2β, which is mainly expressed in muscle cells. In this case, the ubiquitination of filamin B by ASB2β leads to proteasome-dependent degradation (Bello et al., 2009).

In skeletal muscle, E3 ligases also have important regulatory functions in signaling pathways. For example, it was recently found that the ubiquitin ligase Fbxo40 regulates anabolic signals (Shi et al., 2011). Fbxo40 ubiquitinates and affects the degradation of insulin receptor substrate 1 (IRS1), a downstream effector of insulin receptor-mediated signaling. Inhibition of Fbxo40 by RNAi induces hypertrophy in myotubes, and *Fbxo40* knockout mice display bigger muscle fibers (Shi et al., 2011).

Although some E3 ligases involved in muscle protein ubiquitination and breakdown have been identified, very little is known about how ubiquitinated proteins are recognized and delivered to the proteasome. ZNF216 has been identified as an important player in the recognition and delivery of ubiquitinated proteins to the proteasome during muscle atrophy. Interestingly, ZNF216 is upregulated by FoxO transcription factors in atrophying muscles, and ZNF216-deficient mice are partially resistant to muscle loss during denervation. The absence of ZNF216 in muscle leads to the accumulation of polyubiquitinated proteins (Hishiya et al., 2006).

Another important system for extraction and degradation of ubiquitinated proteins from larger structures is the p97/VCP ATPase complex. P97/VCP is induced during denervation, and overexpression of a dominant negative p97/VCP reduces overall proteolysis by the proteasome and lysosome pathways, and blocks the accelerated protein breakdown induced by FoxO3. Interestingly, p97 and its cofactors, Ufd1 and p47, have been found associated with specific myofibrillar proteins, suggesting a role for p97 in extracting ubiquitinated proteins from myofibrils (Piccirillo and Goldberg, 2012).

While a great body of research has focused on the ubiquitination process, little is known about the role of deubiquitination, and its contribution to muscle atrophy. The largest class of deubiquitinating enzymes (DUBs) are ubiquitin-specific proteases (USPs). So far, only two (USP14 and USP19) have been found to be upregulated in atrophying muscles (Combaret et al., 2005; Gomes et al., 2001). Knocking down USP19 in myotubes results in decreased protein degradation and reverts dexamethasone-induced loss of myosin heavy chain (Sundaram et al., 2009).

### 2.3 Proteolysis-dependent regulation of protein synthesis and therapeutic application

**Fig. 2:**
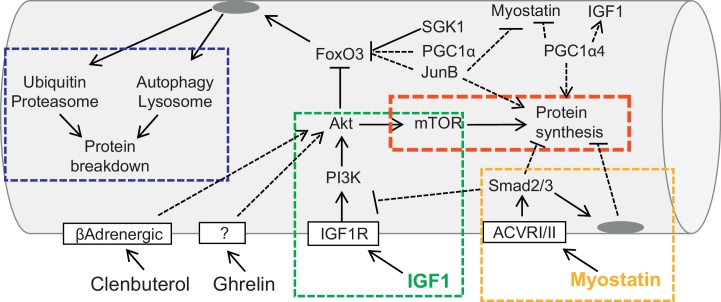
Signaling modules that are important therapeutic targets to counteract muscle wasting. Most of the pathways converge onto a final common pathway centered on Akt-mTOR-FoxO module. The dotted lines point to pathways that are not yet dissected.

Synthesis and degradation of proteins are two processes that are intimately connected. Indeed, most of the above mentioned systems are regulated by pathways that impinge on both synthesis and degradation, so that when protein synthesis is induced degradation is suppressed and vice versa (Fig. 2). This control seems to be a compensatory mechanism to limit the energy expenditure for the production of novel proteins during catabolic conditions. However, in certain situations protein synthesis is increased during muscle atrophy. For instance, in denervated muscles net protein synthesis is increased rather than decreased compared to innervated muscles (Quy et al., 2012). This is because a portion of the amino acids released from protein breakdown stimulate protein synthesis via mTOR, and if this mechanism is blocked, then muscle loss is exacerbated (Quy et al., 2012). The direct action of amino acids on translation plays an important role in the production and activation of stress response pathways, changing metabolism and expression of sarcomeric proteins in order to optimize muscle homeostasis and performance to the new condition. An important example of amino acid-dependent regulation of gene transcription during a catabolic state has recently been described for lysosomal-dependent protein degradation. Nutrients, especially free amino acids, are sensed by the mTOR kinase, which then inhibits autophagy by blocking the formation of the Atg1/ULK1 complex, an important regulatory step for autophagy initiation. The mTORC1 complex is therefore at the center of a variety of cellular process such as protein synthesis, autophagy, ageing, mitochondrial function and energy production. These different actions of mTORC1 are exploited by its localization/recruitment to different cellular compartments. For instance, the Rag GTPase complex, which senses lysosomal amino acids, promotes the localization of mTORC1 at the lysosomal surface. Accumulation of amino acids within the lysosomal lumen generates an activating signal that is transmitted to the Rag GTPases via the vacuolar H^+^-adenosine triphosphatase ATPase (v-ATPase) recruiting mTORC1 to the lysosomes. This mTOR localization initiates the amino acid signaling and protein synthesis (Zoncu et al., 2011). Concomitantly, mTOR also inhibits the Transcription Factor EB (TFEB), a master regulator of lysosome biogenesis (Settembre et al., 2011). Activation of mTORC1 induces phosphorylation and localization of TFEB at the lysosomal membrane, thus inhibiting its transcriptional activity (Settembre et al., 2012). These data indicate that content/activity of the lysosome directly regulates lysosome biogenesis via an mTOR-TFEB axis. The implication of this signaling as it relates specifically to muscle homeostasis has yet to be investigated.

The mechanisms controlling muscle mass have attracted increasing interest in the scientific community due to their potential to address various clinical problems, such as aging, the prognosis of many diseases, quality of life and sports medicine. The results of recent research offer new and exciting perspectives to the field, that will hopefully identify new therapeutic targets and drugs.

Several potentially interesting therapeutic targets have already been identified, although an effective drug that can counteract muscle wasting is not yet clinically available. The most interesting targets belong to the anabolic pathways and the ubiquitin-proteasome system. The Akt signaling and its downstream targets seem to be at the intersection of several different pathways, including β-adrenergic signaling, myostatin and JunB. Moreover, the IGF1-Akt axis is unique in that it controls both protein synthesis and protein degradation. Therefore, IGF-1 analogs might be extremely useful for counteracting muscle loss and weakness. However, the same pathway plays major roles in other biological processes, including cell survival, and in other contexts it can promote tumorigenesis. Thus, the development of a new generation of IGF1 mimetics that specifically activate the Akt pathway in skeletal muscle is a goal for the field. It is important to underline that prolonged inhibition of protein degradation can have a major impact on protein quality control and muscle performance. Such agents could have major drawbacks, such as promoting the accumulation of misfolded or aggregate-prone proteins (Grumati et al., 2010; Masiero et al., 2009).

Recent data identified SGK1 as an important inhibitor of FoxOs and of muscle atrophy. By studying the mechanisms that protect squirrel from muscle wasting during hibernation it was found that SGK1 and not Akt was the critical factor that blocked FoxO activity and muscle atrophy (Andres-Mateos et al., 2013). The fact that SGK1 controls phosphorylation sites of FoxO that are overlapping with those under Akt regulation, is a well establish concept that has been neglected in the last years (Brunet et al., 2001; Calnan and Brunet, 2008). The role of SGK1 must be considered when Akt phosphorylation does not fit with muscle phenotype and with FoxO phosphorylation. Moreover, SGK1 might be a better therapeutic target than Akt, since its action is more important in preventing muscle atrophy than promoting muscle growth.

In fact promoting protein synthesis without acting on protein breakdown may be not sufficient to prevent muscle atrophy and weakness. Indeed in a recent study it was analyzed the role of TSC1 in muscle growth and atrophy by generating muscle-specific TSC1 knockout mice. These animals showed an activation of TORC1 pathway and an increase of protein synthesis but surprisingly, glycolytic muscles were atrophic due to an hyperactivation of FoxO3-dependent atrophy program (Bentzinger et al., 2013). Therefore, just increasing protein synthesis is not sufficient to counteract an enhancement of protein breakdown when an atrophy program is induced.

An alternative approach would consider the development of drugs that activate negative modulators of FoxO transcription factors such as JunB (Raffaello et al., 2010), or PGC-1α (Sandri et al., 2006). FoxOs have been found to interact with PGC-1α, a critical cofactor for mitochondrial biogenesis (Puigserver et al., 2003; Wu et al., 1999). Maintaining high levels of PGC-1α during catabolic conditions (either in transgenic mice or by transfecting adult myofibers) spares muscle mass during denervation, fasting, heart failure, aging and sarcopenia (Geng et al., 2011; Sandri et al., 2006; Wenz et al., 2009). Similar beneficial effects were recently obtained by overexpression of PGC-1β, a homologue of PGC-1α (Brault et al., 2010). The positive action on muscle mass of these cofactors is due to the inhibition of autophagy-lysosome and ubiquitin-proteasome degradation. PGC-1α and PGC-1β reduce protein breakdown by inhibiting the transcriptional activity of FoxO3 and NF-κB, but they do not affect protein synthesis (Brault et al., 2010). Thus, these cofactors prevent the excessive activation of proteolytic systems by inhibiting the action of the pro-atrophy transcription factors without perturbing the translational machinery. However, a novel form of PGC-1α (PGC-1α4), which results from alternative promoter usage and splicing of the primary transcript, has been recently found to be involved in muscle growth, as shown by the finding that mice with skeletal muscle-specific transgenic expression of PGC-1α4 show increased muscle mass and strength (Ruas et al., 2012). PGC-1α4, which is expressed at significant levels in skeletal muscle, is a shorter, truncated form of the previously described PGC-1α, now referred to as PGC-1α1. PGC-1α4 was found to induce IGF1 and repress myostatin, thus promoting hypertrophy and reducing muscle atrophy during cancer or hindlimb suspension. Interestingly, also JunB markedly suppresses myostatin expression in transfected myotubes and decreases the phosphorylation of Smad3, the transcription factor downstream of the myostatin-TGFβ signaling pathway (Raffaello et al., 2010). JunB can block myofiber atrophy of denervated *tibialis anterior* muscles and cultured myotubes induced by FoxO3 overexpression, dexamethasone treatment or starvation. In these conditions, JunB prevents the activation of atrogin-1 and partially of MuRF-1, thereby reducing the increase in overall protein degradation induced by activated FoxO3. Further analysis revealed that JunB does not inhibit FoxO3-mediated activation of the autophagy-lysosome system, but only ubiquitin-proteasome degradation, by inhibiting atrogin-1 and MuRF-1 induction during catabolic conditions. In fact, JunB directly binds FoxO3, thereby preventing its recruitment to the promoters of key atrogenes. Moreover, JunB overexpression is sufficient to induce dramatic hypertrophy of myotubes and of adult muscle. These hypertrophic changes depend on increased protein synthesis, without affecting the basal rate of protein degradation.

Recently the hormone ghrelin was found to prevent muscle wasting (Porporato et al., 2013). Ghrelin is a peptide hormone that stimulates growth hormone (GH) release and positive energy balance through binding to the receptor GHSR-1a. Only acylated ghrelin (AG), but not the unacylated form (UnAG), can bind GHSR-1a; however, UnAG and AG share several GHSR-1a-independent biological activities. Both AG and UnAG inhibited dexamethasone-induced skeletal muscle atrophy and atrogenes expression. Upregulation of circulating UnAG in mice impaired skeletal muscle atrophy induced by either fasting or denervation without stimulating muscle hypertrophy and GHSR-1a-mediated activation of the GH/IGF-1 axis. Importantly, both AG and UnAG seems to activate Akt independently of the receptor GHSR-1a and therefore are acting on an unidentified receptor. Besides the therapeutic properties of AG/UnAG the identification of their receptor in muscle is an important issue for developing novel and more specific drugs.

Beta-adrenergic agonists such as clenbuterol are considered pro-growth and anti-atrophic drugs. Most effects of clenbuterol are mediated by activating Akt-mTOR signaling (Kline et al., 2007), so the concerns associated with IGF1 stimulation can also be applied to β-adrenergic agonists. However a recent report revealed that the β-adrenergic signal might act via a different mechanism (involving a G protein coupled receptor and Gαi2 induceing hypertrophy through inhibition of GSK-3β and activation of p70S6K1) that is independent of the PI3K-Akt axis (Minetti et al., 2011). Notably, the contribution of p70S6K1 and its downstream target S6 to protein synthesis in muscle is uncertain, as S6K1 knockout mice show no impairment of polysome formation, protein synthesis or protein degradation (Mieulet et al., 2007). Therefore, S6 phosphorylation should not be considered a marker of protein synthesis, and we need a better understanding of which mTOR downstream targets are crucial regulators of protein synthesis in order to evaluate the role of this pathway in muscle growth and its potential in therapeutic approaches.

The last category of drug targets is represented by the proteasome system. Proteasome inhibitors have been successfully used to block atrophy in different animal models (Caron et al., 2011; Jamart et al., 2011; Supinski et al., 2009). However, the ubiquitin-proteasome system regulates many relevant biological processes and its prolonged inhibition might be detrimental for muscle cells. Indeed, patients chronically treated with bortezomib, a proteasome inhibitor that was approved by FDA to treat multiple myeloma, display cardiac complications (Enrico et al., 2007). Therefore, more specific approaches that can target the ubiquitin ligases involved in ubiquitination and degradation of sarcomeric proteins should be pursued. Among the different ubiquitin ligases, MuRF1 and TRAF6 seem interesting candidates for developing specific inhibitors. However, ablation of MuRF1 or TRAF6 only partially protects from muscle loss during denervation in mice (Bodine et al., 2001; Paul et al., 2010), indicating that other ubiquitin ligases are also involved in protein degradation. Moreover, it is still unknown whether different muscle atrophic conditions recruit different sets of ubiquitin ligases. Nonetheless, the findings of these last few years have greatly enhanced our knowledge of protein synthesis and degradation in skeletal muscle, and there is increasing hope that it will be possible to develop efficient therapeutic approaches for counteracting muscle wasting in the near future.
